# Use of Bovine Pericardium and Sutureless Biological Glue in Left Ventricular Rupture After Mitral Valve Replacement, Five Years of Follow-up

**DOI:** 10.5935/1678-9741.20150068

**Published:** 2015

**Authors:** Mário Augusto Cray da Costa, Fernando Cesar Laforga, Josué Abrão Maftum, Mateus Gustavo Favaro

**Affiliations:** 1 Universidade Estadual de Ponta Grossa (UEPG), Ponta Grossa, PR, Brazil, and Santa Casa de Misericórdia de Ponta Grossa, Ponta Grossa, PR, Brazil; 2 Santa Casa de Misericórdia de Ponta Grossa, Ponta Grossa, PR, Brazil; 3 Universidade Estadual de Ponta Grossa (UEPG), Ponta Grossa, PR, Brazil

**Keywords:** Postoperative Hemorrhage, Mitral Valve, Cardiovascular Surgical Procedures

## Abstract

Rupture of the left ventricular wall after mitral valve replacement is an infrequent but lethal complication. Reporting correction technique of ventricular rupture with bovine pericardium patch secured with glue and without suturing: a 51 years-old female patient, with double rheumatic mitral lesion, severe stenosis and discrete insufficiency, who had a mitral valve replacement. During surgery, the patient presented a ventricular rupture of the posterior wall (atrioventricular disruption), which was successfully repaired using bovine pericardium with sutureless biological glue over the epicardium of the damaged area. Sixty months after surgery the patient has no symptoms.

**Table:** 

**Abbreviations, acronyms & symbols**	
ECG	= Electrocardiography
ICU	= Intensive care unit
LV	= Left ventricle
NYHA	= New York Heart Association

## INTRODUCTION

Cardiac rupture was first described by William Harvey, in 1647, and, in 1967, mitral valve replacement was referred to in a study of autopsies^[1]^. Its incidence and mortality rates are about 1% and 85%, respectively^[2]^. The etiology of rupture is still not well defined^[3]^.

## PRESENTATION CASE

We present a case of a 51-year-old female patient who has authorized the publication of this case preserving her identification. She had heart failure New York Heart Association (NYHA) class III, electrocardiography (ECG) with atrial flutter.

Echocardiogram identified a marked dilation of the left atrium (65 mm) with extensive internal mural thrombosis; double rheumatic mitral lesion with moderate to severe stenosis (valve area of 1.3 cm^2^ by planimetry); thickened aortic valve with mild regurgitation and global systolic performance of left ventricle (LV) preserved. Coronary angiography was normal.

## TECHNIQUE AND RESULTS

**Fig. 1:**
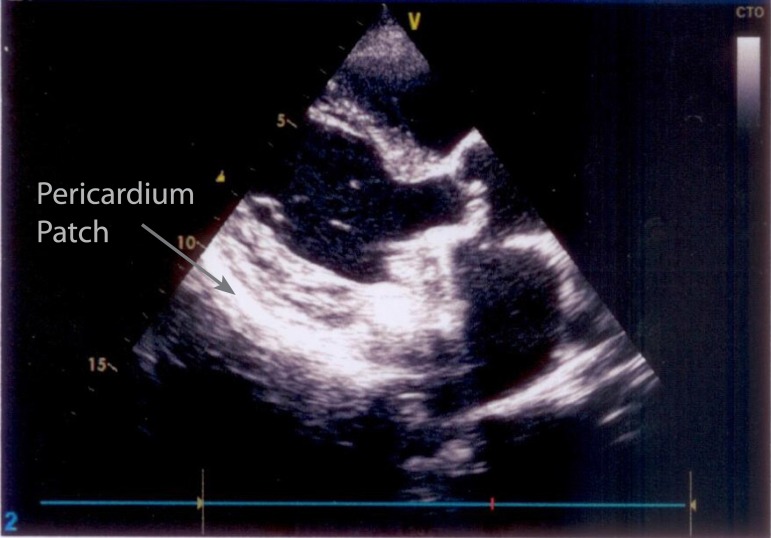
Fixed bovine pericardium patch.

Mitral valve replacement surgery was performed on 7/16/2009 through median sternotomy. The mitral valve had fibrosis with a retraction of both cusps and calcified areas that progressed toward the posterior annulus of the mitral valve, hindering the preservation of tissue in this area of risk. The removal of posterior cusp calcium took the fragility of the posterior ring. A St. Jude 29 mechanical prosthesis was fitted and the pulmonary veins were isolated by cauterization of the endocardium, for the reversal of atrial fibrillation. After decannulation, infiltration of the rear wall by extensive hematoma was observed, and various bleeding points, characteristic of rupture of the rear wall, possibly due to the removal of calcium from the posterior ring that seriously weakened the region. As there was no bleeding yet, just blood infiltration and hemorrhagic diffuse points on musculature, it was opted for an alternative strategy for bleeding correction: a flap of bovine pericardium was immediately fixed with N-butyl-2 cyanoacrylate and methacryloxysulfolane (Glubran^®^) over the area of bleeding, filling the retrocardiac space with absorbable gelatin sponge (Gelfoam^®^) for compression, without the use of sutures (Figure 1). With these measures and the control of blood pressure (mean arterial pressure maintained between 50 and 60 mmHg), bleeding was contained.

After the operation, the patient was referred to the Intensive Care Unit (ICU) under stable hemodynamic conditions. Regarding pressure, mean blood pressure between 50 and 60 mmHg was maintained for 72 hours. The haematocrit at the end of the surgery was 27%, it was 21% at first postoperative day. The patient received two units of packed red blood cells.

The total bleeding was 700 ml. There weren't postoperative complications, no acidosis, the patient was extubated 7 hours after the end of the surgery and she was discharged on the seventh postoperative day with 29% of haematocrit. After 60 months of follow-up, the patient is asymptomatic and control echocardiogram shows good LV function, normal functioning mitral prosthesis, and thickened aortic valve with mild aortic regurgitation. ECG normal with sinus rhythm.

## DISCUSSION

Bovine pericardium preserved in glutaraldehyde is one of the most widely used biological tissues in cardiovascular surgery patches. The use of bovine pericardium in cardiac surgery was introduced in 1972 by Ionescu et al.^[4]^ and popularized by Braile et al.^[5]^. In the present case, it was used externally to the LV posterior wall to stem the bleeding caused by rupture after mitral valve replacement. Roberts & Morrow described the first two cases of ventricular rupture; namely, rupture attributed to the weakness of the wall by the withdrawal of leaflet calcification in one case, and in the other, the perforation of the ventricular wall by excessive withdrawal of the papillary muscle^[1]^.

Patients most at risk are those who are older than 60 or those who are undergoing a mitral valve reoperation.

The occurrence of ventricular rupture after the replacement of the mitral valve is due to several factors^[1]^. It can be caused by improper selection of prosthesis that replaces the valve, especially in elderly patients or female patients with little fibrous rings^[1,2]^. The prosthesis bigger than ring can cause local ischemia. Usually, the prosthesis may depress mitral ring, creating a wall akinesia. These akinetic region protrude out each systole, leading to a stretch of myocardial fibers with overlapping vascular lesions. A hematoma is just formed in the region, raiding the LV wall, resulting in an immediate rupture. Belatedly the presentation could evolve to a ventricular dysfunction or even for an aneurysm^[2]^. Several techniques for ventricular wall repair were described, with or without removal of the prosthesis. The surgeon has to avoid take much calcium or fibrotic tissue at posterior ring. The posterior ring has less fibrous tissue than anterior ring and it is more susceptible to rupture.

Ventricular rupture can be classified as early, delayed, or late if the event occurs at the time of surgery. It is most serious if the event is postoperative (usually in the ICU) or years after surgery^[1,6]^.

Treatment of ventricular free wall rupture is essentially surgical. The first described techniques for the containment of bleeding involved the use of deep points in the myocardium, with the possible complication of circumflex artery injury, as well as the difficulty in supporting the suture due to the fragility of the ventricular cardiac muscle^[6]^. To reduce the possibility of these complications, another technique was described using pericardium patch sutured internally on the ventricular injury^[7]^. In the 1980s, techniques were proposed using Teflon flaps, and subsequently, using biological glue, alternative procedures were allowed to be used without the need for cardiopulmonary bypass. There are reports in the literature of the use of bovine pericardium affixed with biological glue for the treatment of ventricular wall rupture after myocardium infarction^[8]^. There are no reports in the literature about the use of patches fixed with biological glue without suture in cases of rupture after mitral valve replacement, as reported in the present case. This is an alternative treatment that is both safe and effective. That technique can be used in special cases when there isn't massive bleeding. It's essential early diagnosis, before large muscular disruption or infiltration by haematoma. Pericardial fixed with glue can avoid complete rupture of ventricular wall. Considering that other techniques have poor prognosis, that strategies can be an interesting alternative.

## CONCLUSION

We conclude that the use of bovine pericardium fixed with biological glue on the injured area in posterior wall rupture after mitral valve replacement proved to be an effective alternative in the treatment of this highly lethal complication.

**Table:** 

**Authors' roles & responsibilities**	
MACC	Conception and design; final approval of the manuscript
FCL	Design and drawing of the study; implementation of projects/experiments, final approval of the manuscript
JAM	Analysis/interpretation of data; manuscript writing or critical review of its contents; final approval of the manuscript
MGF	Manuscript writing and critical review of its contents
